# LINCE PLUS software for systematic observational studies in sports and health

**DOI:** 10.3758/s13428-021-01642-1

**Published:** 2021-09-21

**Authors:** Alberto Soto-Fernández, Oleguer Camerino, Xavier Iglesias, M. Teresa Anguera, Marta Castañer

**Affiliations:** 1 National Institute of Physical Education of Catalonia (INEFC), University of Lleida, Lleida, Spain; 2 IRBLLEIDA (Lleida Institute for Biomedical Research), University of Lleida, Lleida, Spain; 3 National Institute of Physical Education of Catalonia (INEFC), University of Barcelona, Barcelona, Spain; 4 Faculty of Psychology, Institute of Neurosciences, University of Barcelona, Barcelona, Spain

**Keywords:** Research software, Behavior analysis, Health applications, Sport applications, Systematic observation, Mixed methods

## Abstract

This paper aims to offer a free software program, LINCE PLUS, suitable for systematic observational studies in sports and health, conducted in natural contexts such as training, education or psychology. Using one or several videos simultaneously, different parameters such as behaviors, decision-making or strategies can be analyzed. The software includes several functionalities for studies that researchers need to utilize throughout the observational study process. Collaborative work can be accomplished by using simultaneous videos and multiple observers. The results of all research conducted by LINCE PLUS are offered inside the application in real time, enabling common calculations or including specific analysis with R language without the need for any other external tool. Moreover, LINCE PLUS shows the results of each study with interactive charts or, if needed, it exports the data to specific data analysis software programs (e.g., SAS, Excel, Theme, GSEQ 5, Hoisan). We include examples of sports and health studies that have been conducted with LINCE PLUS to show the suitability of this software program.

## Introduction

The observational methodology "is characterized by high scientific rigor and flexibility throughout its different stages and allows the objective study of spontaneous behavior in natural settings, with no external influence" (Anguera, 1979; Anguera et al., 2017, p. 1). This methodology integrates qualitative and quantitative elements in its QUAL-QUAN-QUAL development stages (Anguera et al., 2020). We take as a starting point the proposal by Creswell and Plano Clark (2007) regarding the *connecting* option about mixing qualitative and quantitative elements, that is, “connecting two datasets by having one build on the other” (p. 7); thus we consider this observational methodology as a *mixed method* itself (Anguera & Hernández-Mendo, 2016). The integration of qualitative and quantitative elements typical of *mixed methods* (O'Cathain et al., 2010; Anguera et al., 2018) is a methodological requirement in research based on systematic observation (Bazeley, 2012, 2016).

This approach to methodological integration has been applied in psychological research on spontaneous behavior in the fields of health (e.g., Casarrubea et al, 2018; Castañer et al., 2017; Puigarnau et al., 2016), physical activity and sport (e.g., Amatria et al., 2017; Fernandes et al., 2020; Fernández-Hermógenes et al., 2021; Gutiérrez-Santiago, 2013; Lapresa et al., 2018; Prieto-Lage et al., 2020; Sastre et al., 2021), and physical education (e.g., Camerino et al., 2019; Castañer et al., 2009, 2011, 2020; Prat et al., 2019; Valero-Valenzuela et al., 2020). Indeed, there are several software programs to conduct visualization and annotation, but with some weakness related to all the necessary features of viewing, coding, analysis and administration of the results, resulting in scattered data and necessitating the export of these results to other complementary software programs (Hernández-Mendo et al., 2014; Love et al., 2019).

For this reason, we have developed a new free and open-source software, LINCE PLUS (Soto-Fernández et al., 2019), optimizing the previous version of LINCE (Gabin et al., 2012). LINCE PLUS allows one to develop all the phases of the observational investigation of spontaneous behavior in an integrative way, featuring (a) a better level of usability, (b) greater simplicity in data processing, (c) the possibility of performing calculations statistics from the platform, (d) the integration of several simultaneous videos, (e) the connection of various devices, and (f) the ability to be used simultaneously by several observers (Anguera et al., 2018; Chacón-Moscoso et al., 2019; Portell et al., 2015).

The objective of this article is to present the characteristics, functionalities and benefits of the LINCE PLUS software program that satisfy the needs of observational studies applied to health and sports.

## LINCE PLUS, a methodological challenge

LINCE PLUS is a desktop application generated in the Java programming language, which incorporates a series of characteristics that allow for collaboration and management of the observational research process through a developed and optimized web interface (Soto-Fernández et al., 2019). This type of application, a hybrid between a desktop and web application, can be installed on any MacOS or Windows 64-bit computer and enables the interconnection of mobile/tablet devices and additional users, facilitating collaborative work and integrating quantitative and qualitative observational record results typical of mixed methods (Anguera et al., 2020).

It allows the calculation of statistical results using the R programming language (Ihaka and Gentleman, 1996), which can be programmed from the web interface or using the RStudio software program to process data in real time. As an integrating element, it allows the visualization of different videos simultaneously in a synchronized way, favoring the registration process and enabling the analysis of behavioral episodes and the representation of results instantly in automatic graphics. This method of data processing simultaneously with the observational record, without the use of external computer applications, simplifies and accelerates the observational research process.

The application and its source code can be downloaded, as research software, on the web (http://www.observesport.com/) or on GitHub (https://observesport.github.io/lince-plus/) platform with information on improvements and incidents.

## LINCE PLUS functionalities

**Fig. 1:**
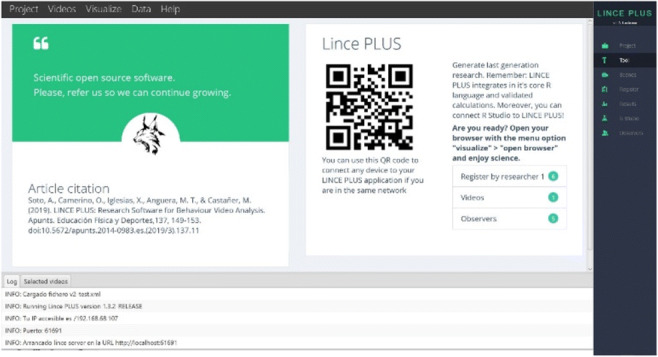
LINCE PLUS desktop application with QR

**Fig. 2:**
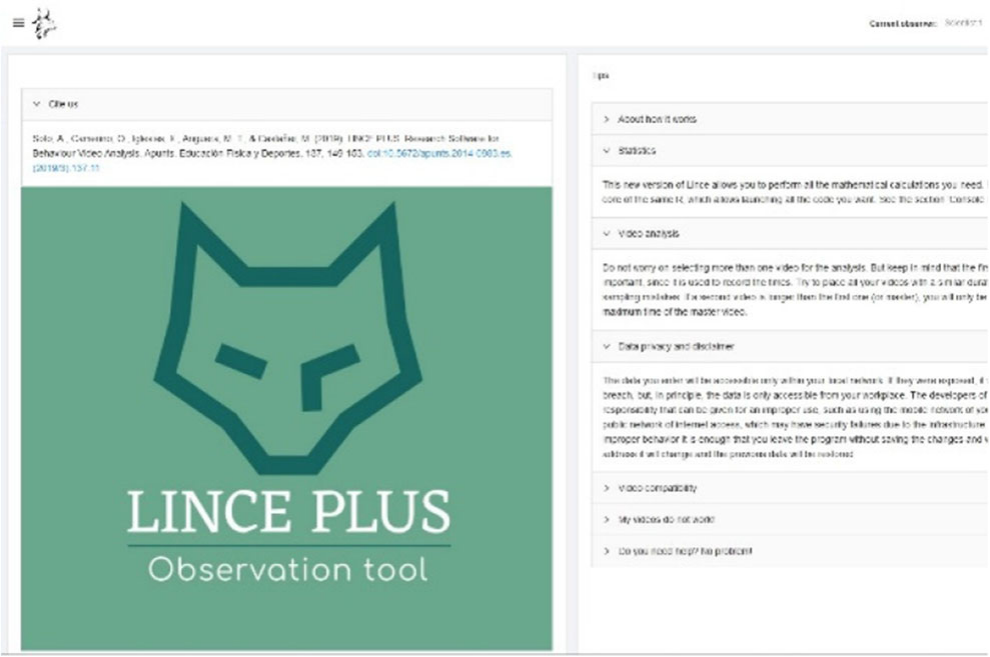
Lince web

The use of web technology requires two components: a desktop application (Lince desktop) in which to load the projects and the images, videos or text materials (Fig. 1), and a web application (Lince web), in which we will create the record and analysis of the research (Fig. 2).

Once the main desktop application (Fig. 1) is executed, we can manage all our project data and selected videos, and integrate our research with other scientific applications such as Theme, Excel or Hoisan. The software does not impose limitations on the number of registered episodes and adapts the data visualization according to the number of criteria that have been designed. Moreover, we can also connect mobile devices and tablets using the QR code or by accessing the web application (Fig. 2) via the “visualize” option, which will use the default browser to access the modules: (a) *Project*, definition of the problem and research design with exploratory qualitative data and initials; (b) *Tool*, construction of an emerging and adequate *ad hoc* observation instrument with facets or dimensions; (c) *Record*, systematization of the record in the form of code matrices; (d) *Results* and *RStudio*, descriptive and quantitative data analysis that will enable the qualitative interpretation of the results; and (e) *Observers*, checking the record of the observers and their agreement.

### Project module: Delimitation of the problem, observational design and observers

We delimit the project by stating the objective of the study and design according to (a) observation units, with idiographic or nomothetic options, (b) temporality, punctual or inter-sessional and intra-sessional follow-up or no intra-sessional follow-up, and (c) dimensionality, of one or more dimensions (Anguera et al., 2011); also, this *Project module* states allows the observers to carry out the study.

### Tool module: Construction of the observation instrument

**Fig. 3:**
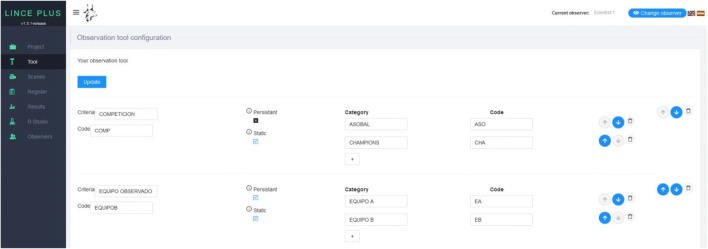
*Tool module*: construction of the LINCE PLUS observation instrument

We will progressively build an observation instrument by displaying the proposed dimensions and structuring them in criteria and emerging categories that will allow us to obtain the first qualitative exploratory records; both in direct observation, through videotaped behaviors, and as indirect, with verbal and/or vocal behavior recorded in audio, texts and images, to progressively translate them into this *Tool module* in quantitative coding (Fig. 3).

### Record module: Visualization, recording and coding of behaviors.

**Fig. 4:**
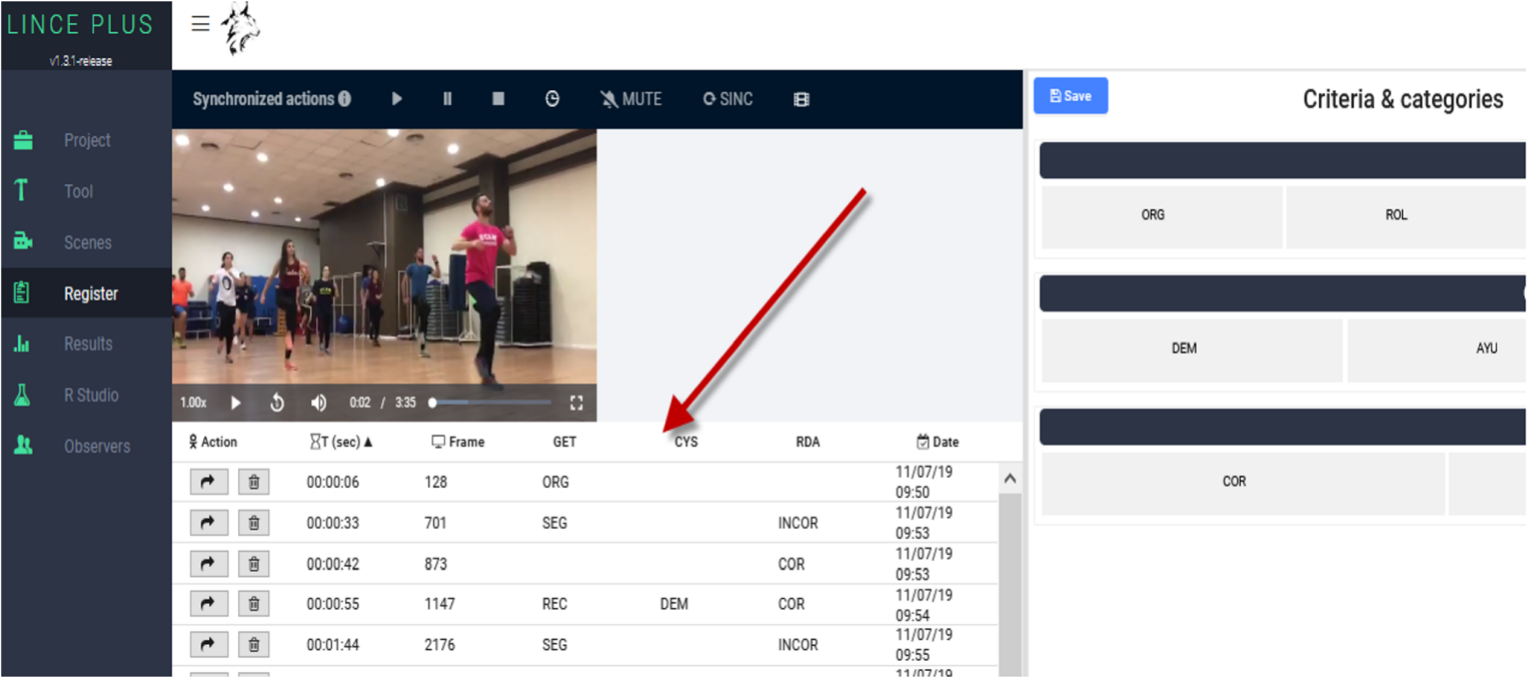
*Record module*: display, record and coding module of LINCE PLUS

Frequency or occurrence, order or sequence, and duration are the fundamental primary parameters of behavioral analysis (Bakeman, 1978). In this *Record module* (Fig. 4), using the previously built observation instrument and the selected images or videos to create the record of the categories that correspond, we will start the recording of the order and frequency of the behavior which the *quantitizing* carries out, converting the previous qualitative record into code matrices, which are then subjected to a robust quantitative analysis, incorporating the duration, mainly from diachronic analysis (Anguera et al., in press).

**Fig. 5:**
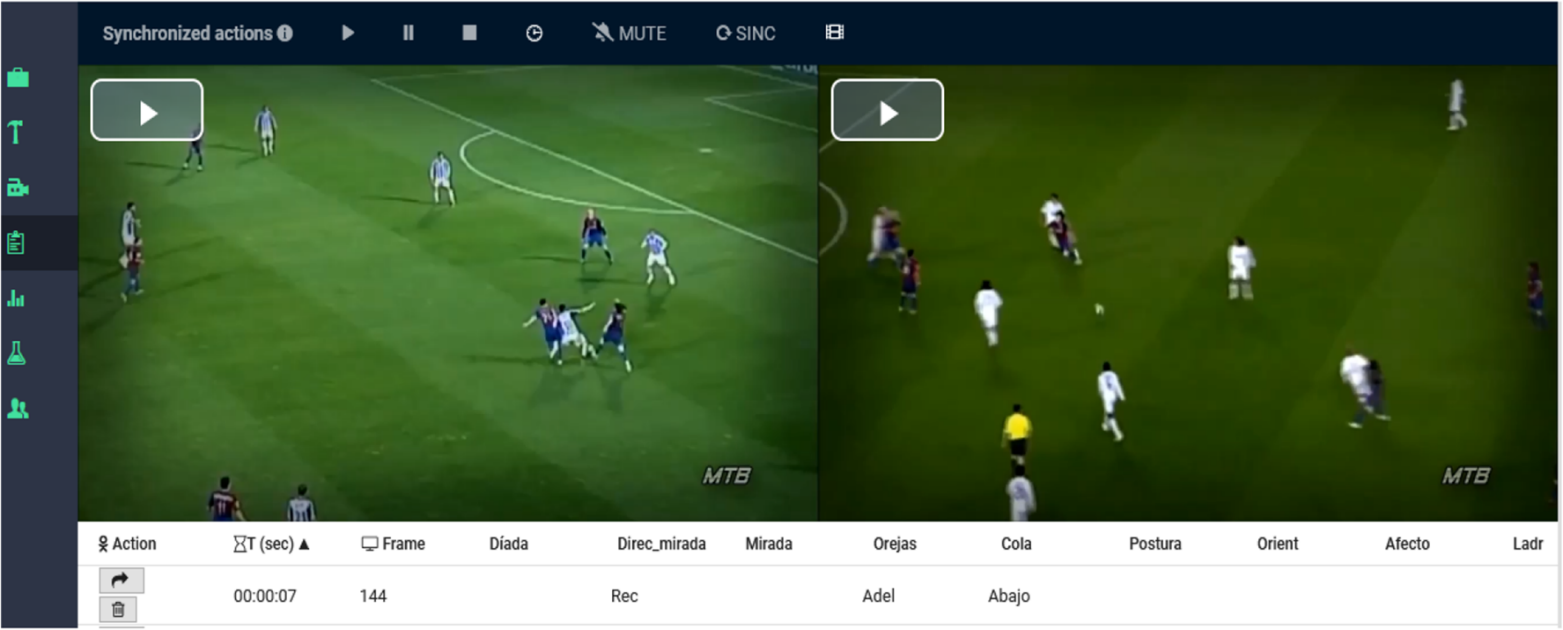
Observation of simultaneous videos in LINCE PLUS

The incorporation of several image and video files in the same project is a step forward which allows one to work with viewing angles and perspectives of images or videos that can be synchronized in the same session from a simultaneous start (Fig. 5).

### Results module and RStudio module: Integrated results analysis

**Fig. 6:**
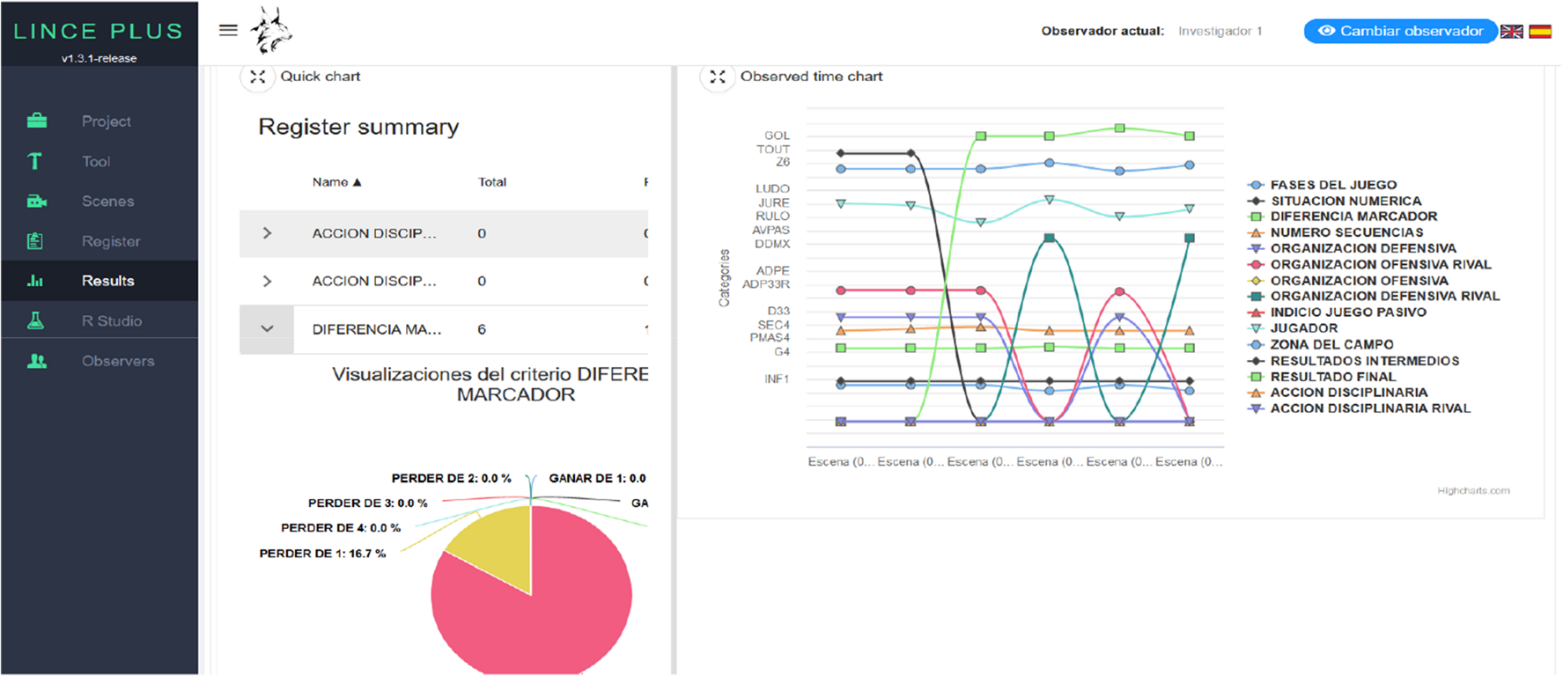
Descriptive results in the *Results module* in LINCE PLUS

**Fig. 7:**
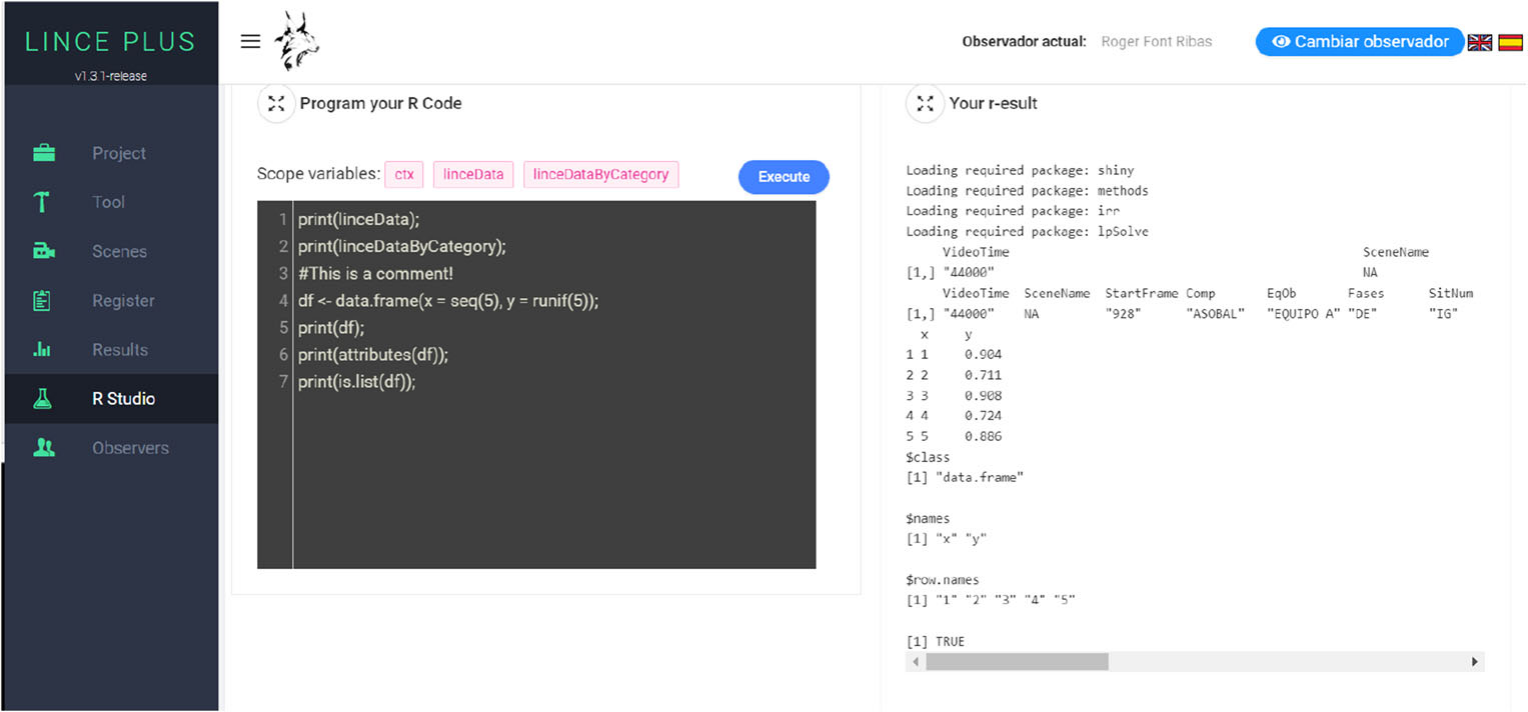
*RStudio module* for executing R code

With the observational record, generated from the annotation and categorization of the images in the previous module, we can obtain results through two procedures and possibilities: externally by exporting them to other software programs (SAS, Microsoft Excel, SDIS-GSEQ, Hoisan, and Theme), selecting the option "data" in the desktop application (Lince desktop) (Fig.1); or internally in a simultaneous and instantaneous way to the development of the study from the following options:

- In the *Results* module (Fig. 6) using the circular sector graphs and/or descriptive or deductive statistics bar diagrams, which give an overview of the distribution of the criteria and categories.
- In the *RStudio* module (Fig. 7) that allows the calculation of specific results, with a statistical analysis applied to the observational record. This *RStudio* module introduces an innovation that increases the capabilities of statistical analysis with the integration of the R programming language in LINCE PLUS, applying a specific and live statistical calculation which complements the previous statistical analysis.

This functionality allows a complete analysis by enabling the statistical engine of the R language to perform calculations in real time, without the need for the observational investigation to have finished, and collaborative work between several analysts, by allowing observation simultaneously with the verification of partial results. We can also incorporate specific R software libraries for the particular and proper analysis of the record. For this, LINCE PLUS has a REST API that allows the RStudio application to be integrated in real time. The instructions to carry out this process are detailed in the LINCE PLUS application itself.

These two approaches or integrations of the R language for the calculation of research results allow the introduction of statistical analysis during the observation phase, anticipating the results of the study without the need to export to other programs, and making it possible to use the observational record in all phases of the investigation. Figure 7 shows how the information processing is carried out immediately from the existing data in the observation record, while another investigator can be recording other episodes.

### Observer’s module: Registration of several observers and their concordance

Regarding the calculation of the agreement index between the different observers involved in the observation, we have arranged a specific section through the Observers module.

**Fig. 8:**
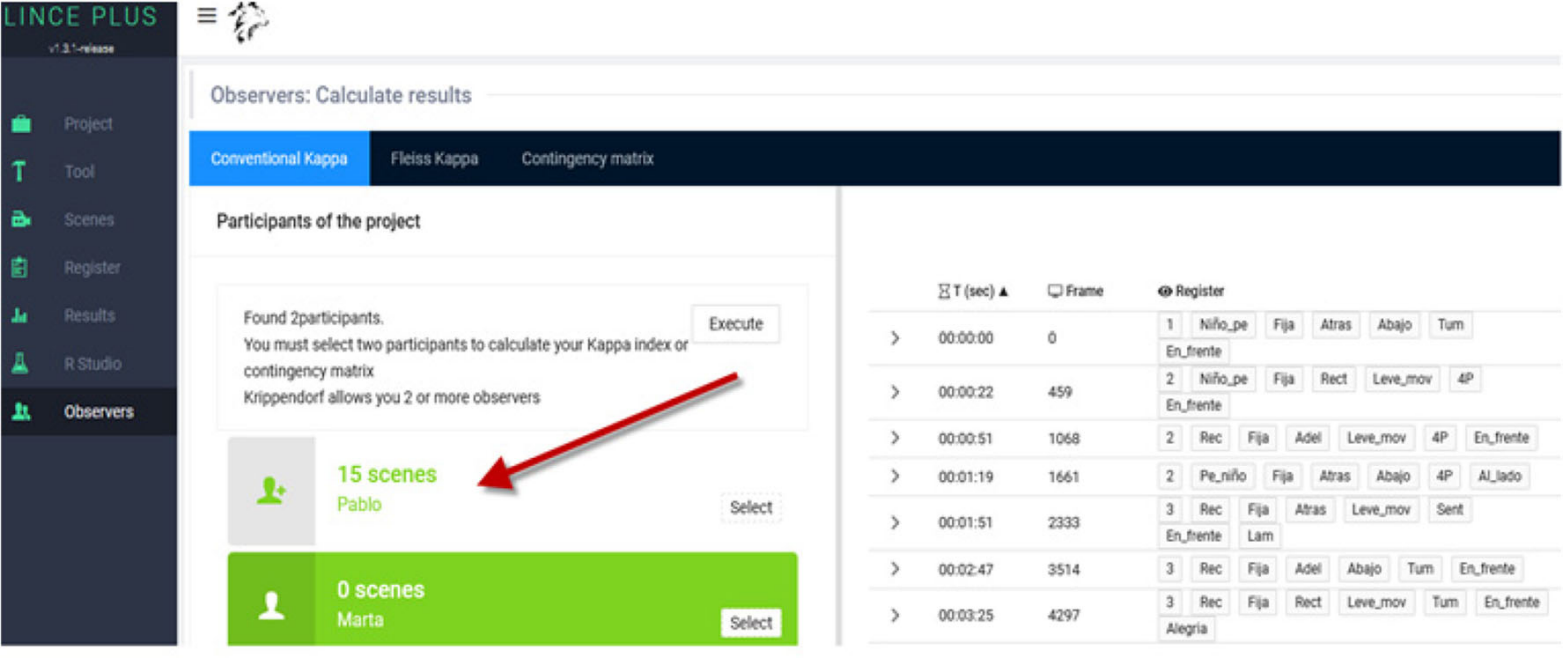
*Observers module*: records between observers of LINCE PLUS

Since it is a web application, it allows the participation of several observers, configured in the same project, allowing the information record to be segmented in episodes that occur on a video recording or using a timer to allow the recording of live information. The management of several observers in this Observers module makes it possible to review the record of each observer at any time (Fig. 8).

**Fig. 9:**
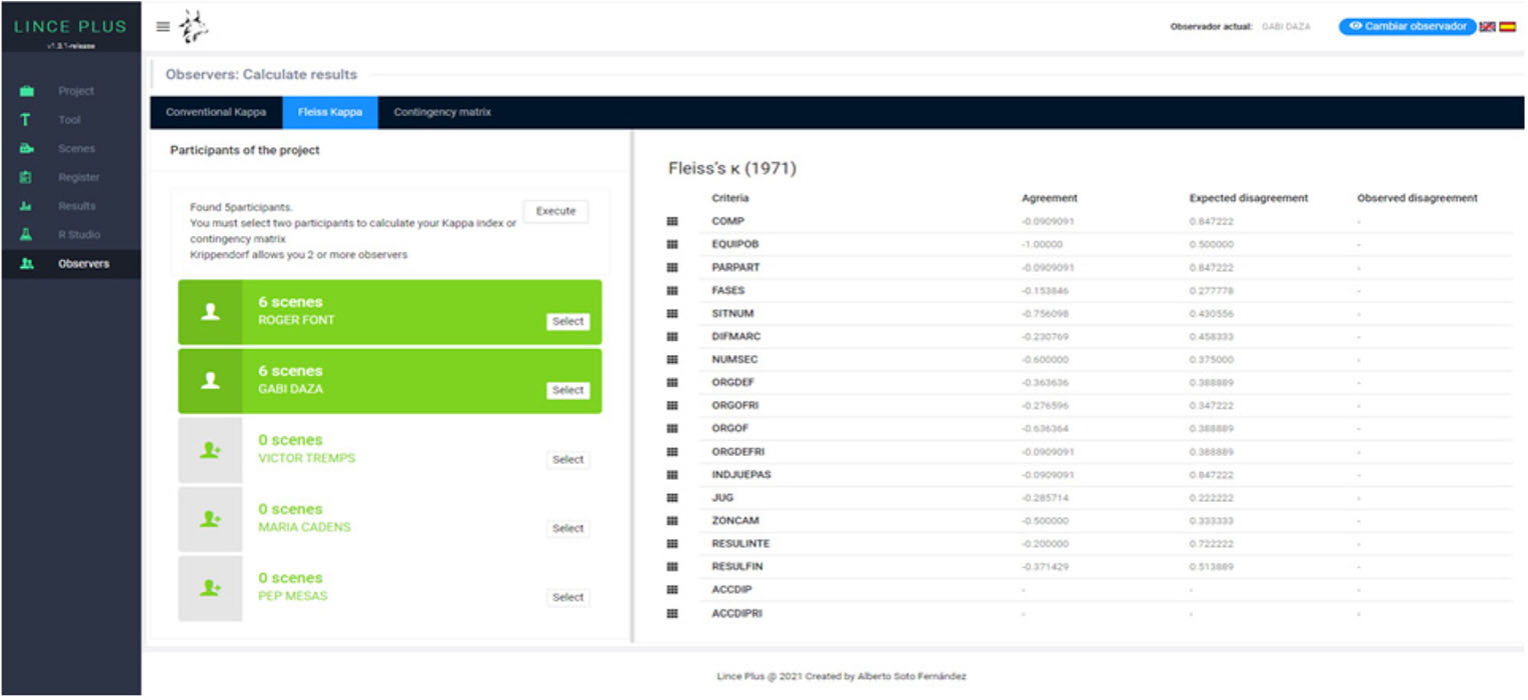
Calculation of the agreement index between various observers in LINCE PLUS

The inclusion of several observers in the same project requires a control of the data quality through inter-observer agreement (from different observers in the same sessions), which can be extended to intra-observer agreement (from the same observer in different sessions). LINCE PLUS includes the calculation at one click for the kappa index by Cohen (1960), kappa index by Fleiss (1971) and Krippendorff index (2019). The agreement via Cohen’s kappa coefficient is calculated from order and duration parameters (Bakeman, 1978; Bakeman et al., 1996), and does the alignment (Quera et al., 2007). These calculi are done thanks to the integration of the DKPro library (Artstein & Poesio, 2008; Meyer et al., 2014) in the web interface, whose calculation can be carried out without exporting the information to other applications (Fig. 9).

## LINCE PLUS features

### Observation instrument versatility

The configuration of the different dimensions or response levels using LINCE PLUS allows the following options:

- Incorporation of free text fields, allowing the introduction of codes corresponding to visually perceptible behaviors (direct observation) and codes corresponding to textual units (indirect observation) seamlessly
- Establishment of persistent criteria or dimensions that do not vary until the researcher deems it appropriate
- Preparation of a field format instrument with dimensions that are displayed in a hierarchical system of expandable codes of conduct.

The generation of the observation instrument in such a versatile way with the Tool module (Fig. 3) of the LINCE PLUS software program is adaptable to the needs of observational studies, facilitating the recording of emerging behaviors of great complexity and quantity.

### Collaborative work

LINCE PLUS allows the live recording of sessions, using mobile devices, and collaborative work between observers connected from their devices. This feature does not require having the application installed and is made possible through the QR code generated at the beginning of each project in the desktop application (Lince desktop) that enables this device connectivity, as long as they are in the same private local network (Fig. 1), a factor that allows data privacy.

### Episode sampling

**Fig. 10:**
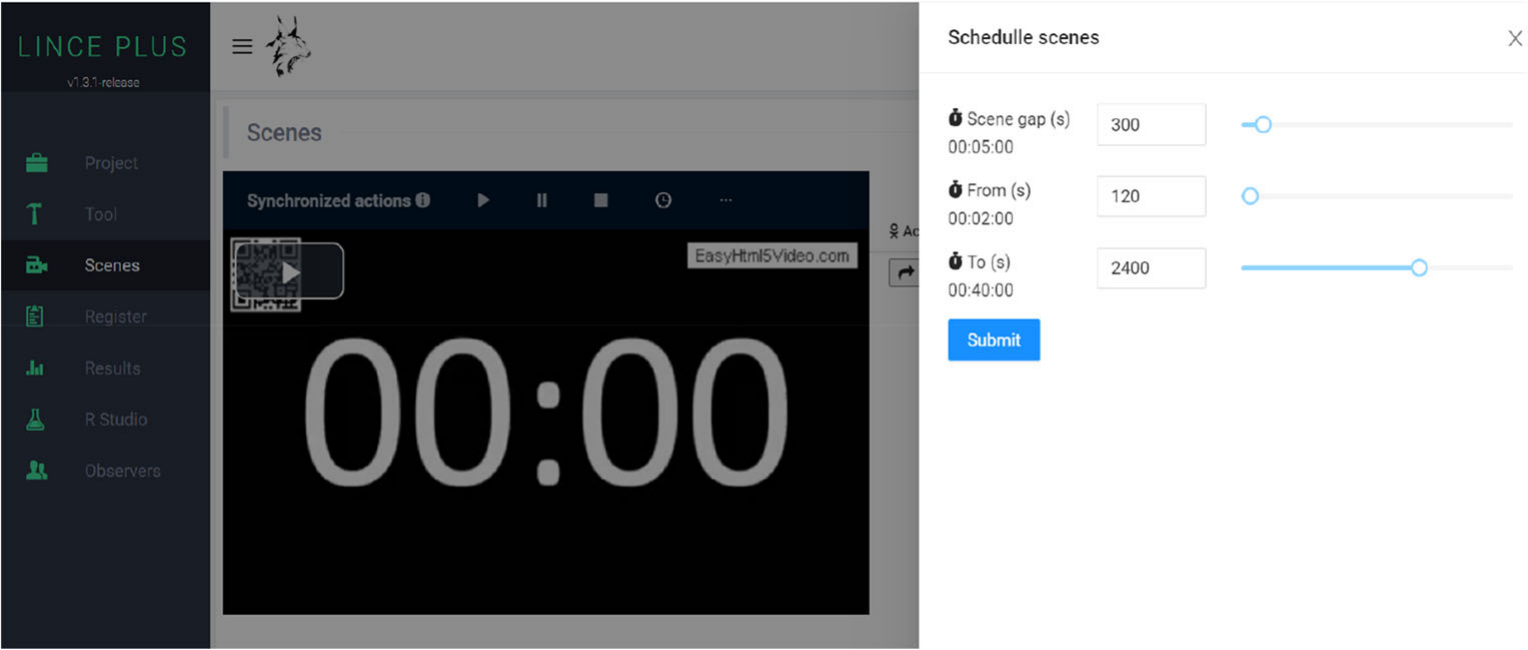
Generation of automatic episodes through the LINCE PLUS *Scenes module*

We must bear in mind that one of the essential characteristics of an observational follow-up study, when it is not possible to record all the episodes, is the performance of a random sampling of episodes (Mehl & Robbins, 2012; Stone et al., 2007). LINCE PLUS allows the automated generation of episodes generating an automatic record per unit of time. To achieve this, the *Scenes* module assistant offers the possibility of generating episodes at regular intervals of time within the maximum video duration (Fig. 10).

## Technological overview

The application consists of an innovative concept that is presented under a traditional desktop application but that nevertheless generates a web server at each startup and that changes its location to facilitate privacy.

This server includes a whole series of innovative concepts from several years of work in which various proofs of concept, prototypes and integrations of libraries and *frameworks* have been carried out. It currently presents the inclusion of Spring Boot in a Java context and is integrated with an application based on JavaFX, which allows the integration of any external application in real time from a REST API. This server also has streaming features and includes the installation of the FFmpeg libraries to automatically convert any selected video to a format compatible with web standards, such as MP4, enabling the backup of the information every 2 minutes and also allowing its execution in Windows and MacOS environments. By displaying web behavior, any device is allowed access via a QR code and includes a streaming server with features of *adaptive streaming* to reduce the size of the information sent, creating an innovative user experience.

On the other hand, in terms of the web section and connected devices, we have generated several development models. The latest model is developed in React and with a design based on Ant-Design under Lerna and monorepo architecture. The persistence of the components has followed a mixed flow from React-redux and React-saga, also integrating prototypes of interconnectivity of Bluetooth low-energy (BLE) devices from the experimental function of interconnection via the web—functionality that will be available in future versions.

In the future, we hope to develop the mobile application with React Native for the integration of more BLE devices and the inclusion of ANT+ devices in a transparent way for the user and, if the community offers its support, the evolution of the platform to a pure web environment, without the need to install updates for each user or the loss of information caused by the loss of files.

## Discussion and conclusion

LINCE PLUS is a tool suitable for any observational design that allows the recording of behavioral observation, live or deferred, from recordings, introducing the possibility of performing this recording with several simultaneous observers and with various connected devices. This incorporation of collaborative work in real time for the research work allows the records of the same episodes, duly contrasted in their agreement, to be analyzed in the application itself or to export the data to other software programs.

The use of the application has grown exponentially in its first year and it is hoped that it will facilitate research work and the generation of studies and publications. (Camerino et al, 2019; Castañer et al., 2020; Prat et al., 2019; Valero-Valenzuela et al., 2020). We believe that the application can make relevant contributions to the research work of behavioral observation in the field of health and sports, as in other areas of human social interaction behavior.

We consider that the development of the LINCE PLUS application has had a very positive result as a tool for integrating knowledge. This result is the outcome of a long and intense development which integrates the following characteristics:

A web interface focused on user experience has been developed and optimized for easy installation and use. The application is starting to be used in several countries.

The application is developed from highly consolidated technology in the computer field and incorporates a web interface that facilitates its integration into the research process. This technology resides in the Java programming language and integrates the use of the Spring framework, R, React, Ant-Design and more under a context that allows it to generate a web server that communicates the data through a REST API.

The introduction of quantitative parameters from physical measurement and incorporation of wearable devices is a factor of great interest. The application is capable of recording the observed heart rate and kinesthetic movements, and will allow the progressive introduction of any type of device that is connectable by Bluetooth. This feature is in full development thanks to Suunto Movesense devices.

We have developed proofs of concept that allow the introduction of artificial intelligence analysis in the video. In the tests carried out, we have designed a model for the detection of human movement and identification of the body segments that allows us to detect the angles of the human body in movement.

The direct connection of the RStudio application to LINCE PLUS is an aspect of great interest and, on the other hand, the execution of R code within the module of our application for the calculation of results allows an unprecedented calculation power that must be known by the research community to facilitate its evolution.

In sum, LINCE PLUS is the result of intense and extensive work of several years that requires constant evolution. Hence, we consider that our project is of great interest and that it has a great future ahead of it, so we hope to continue its development and offer continuous improvements for the research field.

## Limitations and future perspectives

The improvement of LINCE PLUS lies with a few developers, and as it is the fruit of an innovative prototype, which is offered for free as an open-source project, it involves continuous effort that requires the support of the community. Thus, we need constant and greater support from the community to improve the software related to all its functionalities, as described below.

We acknowledge the limitations of LINCE PLUS and how we plan to overcome them:

- The development of a web portal that allows all users to connect without having to install any software or carry out any updates. This web portal would be a knowledge base for the research field and should be able to perform the same functions as LINCE PLUS.
- The development of mobile applications for iPhone and Android. This mobile application could link the data from the sensors and connect them with the web portal, generating a unique research ecosystem.
- Live video-recording will be possible, a factor that would allow the inclusion and synchronization of all the above information and its analysis.
- Improving the support for the community and language translation. Although it supports any language, the translations need to be adapted. Moreover, the support for users is reduced due to a limited budget for the project.
